# Unexpected Lattice-Tip Catheter Overheating During Pulsed Electric Field Ablation Near Metal Implants

**DOI:** 10.1016/j.jaccas.2026.108467

**Published:** 2026-05-22

**Authors:** Kazuto Hayasaka, Predrag Stojadinović, Petr Peichl, Dan Wichterle, Nicoletta Ventrella, Josef Marek, Peter Štiavnický, Jana Hašková, Josef Kautzner

**Affiliations:** Institute for Clinical and Experimental Medicine (IKEM), Department of Cardiology, Prague, Czechia

**Keywords:** Amplatzer septal occluder, AtriClip, lattice-tip catheter, pulsed electric field ablation, Sapien valve

## Abstract

The energy dynamics of pulsed electric field (PEF) ablation near metallic cardiac implants during clinical procedures remains poorly understood. We report 3 cases in which focal PEF ablation using a lattice-tip catheter (Sphere-9) was performed adjacent to an Amplatzer septal occluder, an AtriClip, and a Sapien valve. All cases showed unexpected elevations in catheter temperature (up to 61 °C, 68 °C, and 53 °C), without impedance change or excessive bubble formation on intracardiac echocardiography. Careful real-time monitoring of catheter temperature is warranted, and intracardiac echocardiography may provide additional information when performing PEF energy delivery near metallic devices.

## Visual Summary

**Figure:**
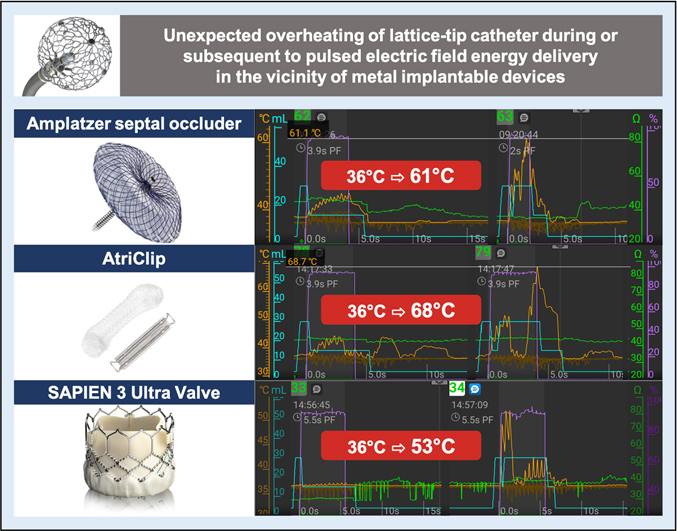

Pulsed electric field (PEF) ablation is increasingly used to treat different cardiac arrhythmias, with high efficacy and a favorable safety profile. Preclinical studies have suggested that PEF energy delivery in proximity to metallic implantable devices may cause electrical interactions, including visible arcing and heating.^1^^,^^2^ However, to our knowledge this phenomenon has not been described in clinical practice. We report 3 cases of unexpected elevation in the lattice-tip catheter temperature during or shortly after PEF delivered in proximity to metallic cardiac implants. Although no overt complications occurred, these observations warrant caution and further investigation of the underlying mechanisms and clinical implications.

## Case 1

### Patient history

A 64-year-old man with a prior transcatheter atrial septal defect closure using a 34-mm Amplatzer septal occluder (Abbott Laboratories) 22 years ago, was scheduled for catheter ablation for symptomatic episodes of atrial fibrillation and atrial flutter.

### Procedure and follow-up

**Figure 1:**
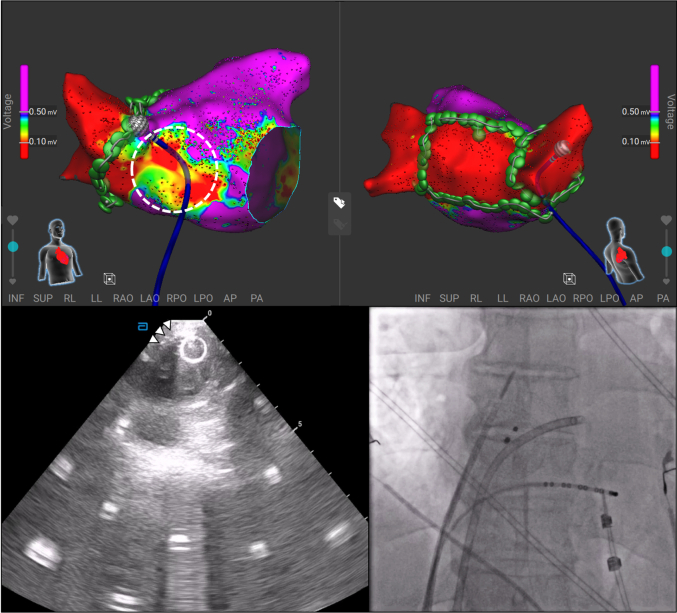
Catheter Position Associated With Temperature Rise and Corresponding Temperature Change (Case 1) During an application near the right superior pulmonary vein, the catheter temperature increased to 61 °C. (Top Left) The white dotted line indicates the estimated location of the occluder. (Bottom Left) On intracardiac echocardiography, the lattice-tip catheter was in proximity to the occluder (arrowheads), and no abnormal bubble stream was observed during the application. (Bottom Right) Left atrial access was successfully achieved by traversing an occluder using a steerable sheath.

Catheter ablation was performed under general anesthesia. After obtaining venous access, intracardiac echocardiography (ICE) confirmed no residual fossa ovalis suitable for transseptal puncture. A steerable sheath (Agilis NxT, Abbott) and a mechanical transseptal needle (BRK XS, Abbott) were used to penetrate the inferior portion of the occluder under the ICE guidance. The device mesh was traversed with the needle, and the sheath was then advanced into the left atrium (LA) over a guidewire (Figure 1). LA mapping with a lattice-tip catheter (Sphere-9 catheter with Affera mapping and ablation system, Medtronic) demonstrated low-voltage areas on the posterior wall. Therefore, in addition to bilateral pulmonary vein isolation, posterior wall isolation was performed, both using PEF energy.

During isolation of the anterior aspect of the right superior pulmonary vein, energy delivery was required in proximity (estimated <2 mm) to the occluder. The catheter temperature increased from a baseline of approximately 36 °C to 61 °C (ΔT: 25 °C), and the application was interrupted at 2 seconds by a temperature limit alert (Figure 1). The temperature rise was not accompanied by impedance change. ICE showed neither an abnormal bubble stream nor a new hyperechogenicity suggestive of unexpected tissue overheating. Right inferior pulmonary vein isolation and posterior wall isolation were completed using PEF ablation without substantial increases in the catheter temperature. The following day, transthoracic echocardiography with color Doppler demonstrated a micro-shunt flow from the LA to the right atrium, consistent with the puncture site and no occluder malposition.

## Case 2

### Patient history

A 65-year-old man underwent coronary artery bypass grafting for ischemic cardiomyopathy 1 year ago. Because of a prior history of atrial fibrillation requiring electrical cardioversion, concomitant pulmonary vein isolation by bipolar radiofrequency ablation (RFA) using Isolator Synergy clamps (AtriCure) and left atrial appendage ligation with a 45-mm AtriClip (AtriCure) were performed at the time of surgery. He later experienced recurrent persistent atrial tachycardia, and catheter ablation was indicated.

### Procedure and follow-up

Under general anesthesia, venous access was obtained, and transseptal puncture was performed under ICE guidance to access the LA. Mapping with the lattice-tip catheter confirmed a perimitral flutter. Because a broad low-voltage area was present on the anterior LA wall, an anterior line was created by RFA from the mitral annulus toward the right superior pulmonary vein, which resulted in arrhythmia termination.

**Figure 2:**
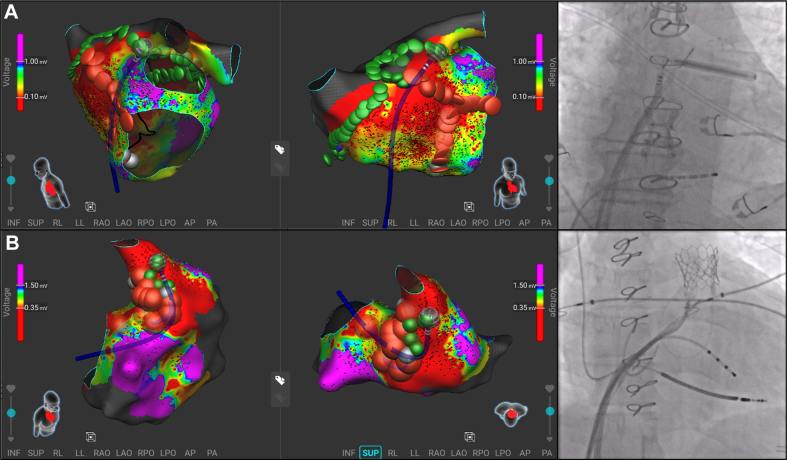
Catheter Position Associated With Temperature Rise and Corresponding Temperature Change (Cases 2 and 3) (A) Case 2: A temperature rise to 68 °C without a concomitant impedance change was observed during an application at the ridge between the left superior pulmonary vein and the left atrial appendage. On fluoroscopy, the catheter position where the temperature rise occurred was close to the AtriClip. (B) Case 3: During a pulsed-field energy application delivered immediately beneath the pulmonary Sapien valve, the catheter temperature increased to 53 °C without a concomitant change in impedance. On fluoroscopy, the Sphere-9 catheter was positioned directly beneath the Sapien valve.

Subsequently, a box isolation of the posterior wall was performed using PEF ablation. During this phase, the catheter temperature increased from a baseline of approximately 36 °C to 68 °C (ΔT: 32 °C), without a marked impedance drop or interruption in application (Figure 2A). On fluoroscopy (anteroposterior view) and the Affera map, the lattice-tip catheter positioned on the anterosuperior aspect of the left superior pulmonary vein ostium was in proximity to the AtriClip. ICE demonstrated neither abnormal bubble formation nor a new hyperechogenic area suggestive of tissue injury due to overheating. Subsequent applications were completed without further catheter temperature elevations, and isolation was successfully achieved.

## Case 3

### Patient history

A 32-year-old man had a history of double-outlet right ventricle, ventricular septal defect, and pulmonary valve stenosis. In 1993, he underwent intraventricular tunnel repair (cavopulmonary shunt), pulmonary valvotomy, ventricular septal defect closure, and right ventricular outflow tract pericardial patch reconstruction. He subsequently underwent pulmonary valve replacement with a 27-mm Epic bioprosthesis (St Jude Medical) and tricuspid valve repair in 2010. Four years ago, he underwent valve-in-valve transcatheter therapy with a 26-mm Sapien 3 Ultra valve (Edwards Lifesciences). He later developed recurrent right ventricular outflow tract ventricular tachycardia (VT) and experienced recurrence despite 2 prior ablation procedures; therefore, repeat ablation was indicated.

### Procedure and follow-up

Under general anesthesia, venous access was obtained. Clinical VT was induced with programmed stimulation from the right ventricular apex. Activation mapping identified the earliest activation site immediately below the pulmonary Sapien valve. VT was terminated with a radiofrequency application at this site, followed by 10 additional radiofrequency applications for substrate homogenization (Figure 2B). However, VT remained inducible, and the earliest activation site was confirmed to have shifted inferiorly. As VT did not terminate with further radiofrequency delivery, PEF applications were carefully delivered around the earliest site, resulting in VT termination, elimination of local electrograms, and VT noninducibility. During the PEF application near the valve (estimated <2 mm), the catheter temperature increased from 36 °C to 53 °C (ΔT: 17 °C) (Figure 2B), without a marked impedance change, obvious bubble formation, or new hyperechogenic area on ICE. The immediately preceding PEF application at a more inferior location, farther from the valve, did not show a similar temperature rise. In addition, ICE did not demonstrate deep catheter embedding or excessive tissue contact at this site. Postprocedural ICE showed no pericardial effusion or valvular damage.

## Discussion

In this case series, we report to our knowledge the first clinical observations of unexpected overheating of the lattice-tip catheter during PEF ablation in proximity to metallic cardiac implants, including an Amplatzer septal occluder, an AtriClip, and a Sapien prosthetic valve. The catheter temperature increased markedly (ΔT: 17-32 °C) compared with usual applications, without a marked impedance change or abnormal bubble formation on ICE, and no overt clinical complications occurred.

In vitro studies have demonstrated that delivering PEF energy near metallic structures can distort the electric-field distribution and, in some cases, result in visible arcing. Even in the absence of direct electrode-metal contact, in silico modeling studies involving metallic intracoronary stents have suggested that a nearby conductive structure can alter electric-field boundary conditions, locally concentrate current density, and potentially lead to focal heating in the intervening tissue. Although these findings are derived from epicardial models using intracoronary stents, they support the broader concept that the metallic nature of adjacent structures may influence local electric-field distribution, even if not directly generalizable to endocardial lattice-tip catheter ablation.^3^ Campos-Villarreal et al^1^^,^^2^ reported visually observed arcing during PEF application performed in direct contact with a left atrial appendage occlusion device, highlighting the potential for metal–electrode interactions under experimental conditions.

In our case series, unexpected temperature elevations of the lattice-tip catheter were observed during or shortly after PEF delivery in proximity to metallic implants, without visible signs of arcing (ie, no abnormal bubble stream) and without marked impedance change. In contrast to prior experimental reports, the metallic devices in our patients had been implanted long before ablation and were therefore likely endothelialized; moreover, the AtriClip is an epicardial device, making direct endocardial contact unlikely. Alternative explanations, such as catheter wedging with reduced convective cooling or irrigation-system malfunction, appear less likely given the anatomical locations, the normal temperature behavior of adjacent applications, and the absence of widespread abnormalities under identical conditions.

Temperature sensing is unique to the lattice-tip catheter, as it is designed to deliver both PEF and radiofrequency energy. In contrast, most currently available PEF tools lack temperature sensing capability. Consequently, when PEF is delivered in proximity to metallic objects, energy delivery may not be automatically interrupted with such tools, or it may only be aborted after a significant temperature rise has already occurred. Flat impedance dynamics during ablation energy delivery, with an excessive rise in temperature, can be explained by the method of impedance measurement, which is performed between a large spherical (9-mm) catheter tip and return electrodes. Such a configuration does not reflect the local impedance change at the catheter-tissue interface.

Indeed, a recent report described esophageal injury during PEF energy delivery using a pentaspline catheter in proximity to an Amulet device (Abbott), which was likely related to thermal overheating caused by abnormal PEF delivery, as multiple applications were aborted.^4^ Another report demonstrated that aborted PEF energy deliveries were associated with abnormal bubble formation detected on ICE, further suggesting substantial local temperature increases.^5^

Although no overt complications occurred in our small series, these findings warrant particular caution. Direct contact between the lattice-tip catheter and metallic implants should be avoided whenever possible; if proximity is unavoidable, switching to radiofrequency energy at a distance from vulnerable structures may be considered, as limited, small-scale reports suggest that RFA can be performed feasibly in selected patients with intracardiac implantable devices, such as septal occluders and prosthetic valves.^6^^,^^7^ Further studies are needed to clarify the mechanisms, frequency, and clinical implications of this phenomenon. These observations are particularly relevant in the era of concomitant PEF ablation and left atrial appendage occlusion, as the number of such combined procedures is increasing. As a result, PEF delivery near metallic implants is becoming more common, underscoring the need for heightened awareness and procedural caution.

**Visual Summary:**
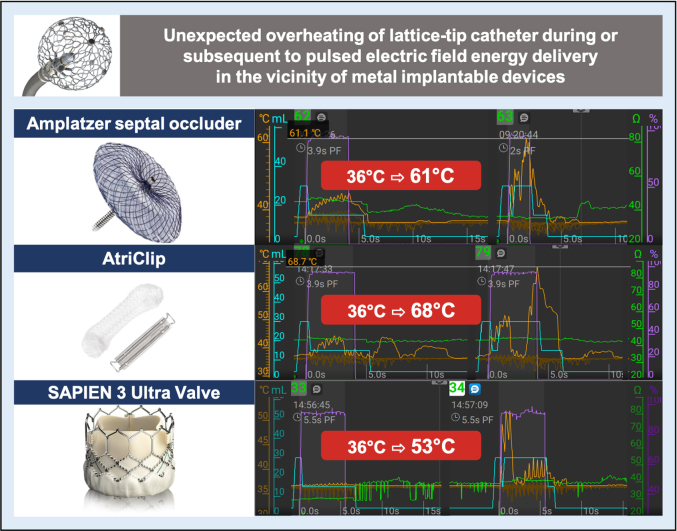
Unexpected Overheating of the Lattice-Tip Catheter During or After Pulsed Electric Field Energy Delivery in the Vicinity of Metal Implantable Devices

## Conclusions

PEF ablation near metallic devices may be associated with unexpected elevations in catheter temperature, resulting in application interruption and potential tissue overheating. In this context, vigilant real-time monitoring of catheter temperature and impedance is warranted to enhance procedural safety. Further studies, including prospective investigations, are needed to better define the relationship between device proximity and temperature elevation, as well as to evaluate peridevice tissue effects using postprocedural imaging.

## Funding Support and Author Disclosures

This study was supported by the project National Institute for Research of Metabolic and Cardiovascular Diseases (Programme EXCELES, Project No. LX22NPO5104), funded by the European Union–Next Generation EU. This work was also funded by the Ministry of Health, Czechia, for the development of research organization 00023001 (IKEM, Prague, Czechia) (institutional support). Dr Peichl has received speaker honoraria from St Jude Medical (Abbott), Medtronic, Boston Scientific, and Biosense Webster. Dr Wichterle has received consulting fees from Johnson & Johnson MedTech. Dr Kautzner reports personal fees from Biosense Webster, Boston Scientific, GE Healthcare, Medtronic, and St Jude Medical (Abbott) for participation in scientific advisory boards, and has received speaker honoraria from Biosense Webster, Biotronik, Boston Scientific, Medtronic, ProMed CS, St Jude Medical (Abbott), and Viatris. All other authors have reported that they have no relationships relevant to the contents of this paper to disclose.

**Take-Home Message**

• Pulsed electric field ablation near metallic cardiac implants, such as an Amplatzer septal occluder, an AtriClip, or a Sapien valve, may result in unexpected elevations in catheter temperature without corresponding impedance changes or clear intracardiac echocardiography findings. Nearby conductive structures may alter local electric-field characteristics even without direct catheter contact.
• When pulsed electric field ablation must be delivered in such settings, careful real-time temperature monitoring is warranted, and intracardiac echocardiography may provide complementary information when abnormal findings are present.
